# Supplementary material for: The interactome of intact mitochondria by cross-linking mass spectrometry provides evidence for coexisting respiratory supercomplexes
Source: Mol Cell Proteomics. 2017 Dec 8;17(2):216–32. doi: 10.1074/mcp.RA117.000470 (PMC5795388; doi:10.1074/mcp.RA117.000470)
Supplement: Supplemental Data [file supp_RA117.000470_133922_0_supp_23978_fzffwf.zip › spectra_annotation/mito_spectra_annotation/175-1-14-1-13-1.pdf]

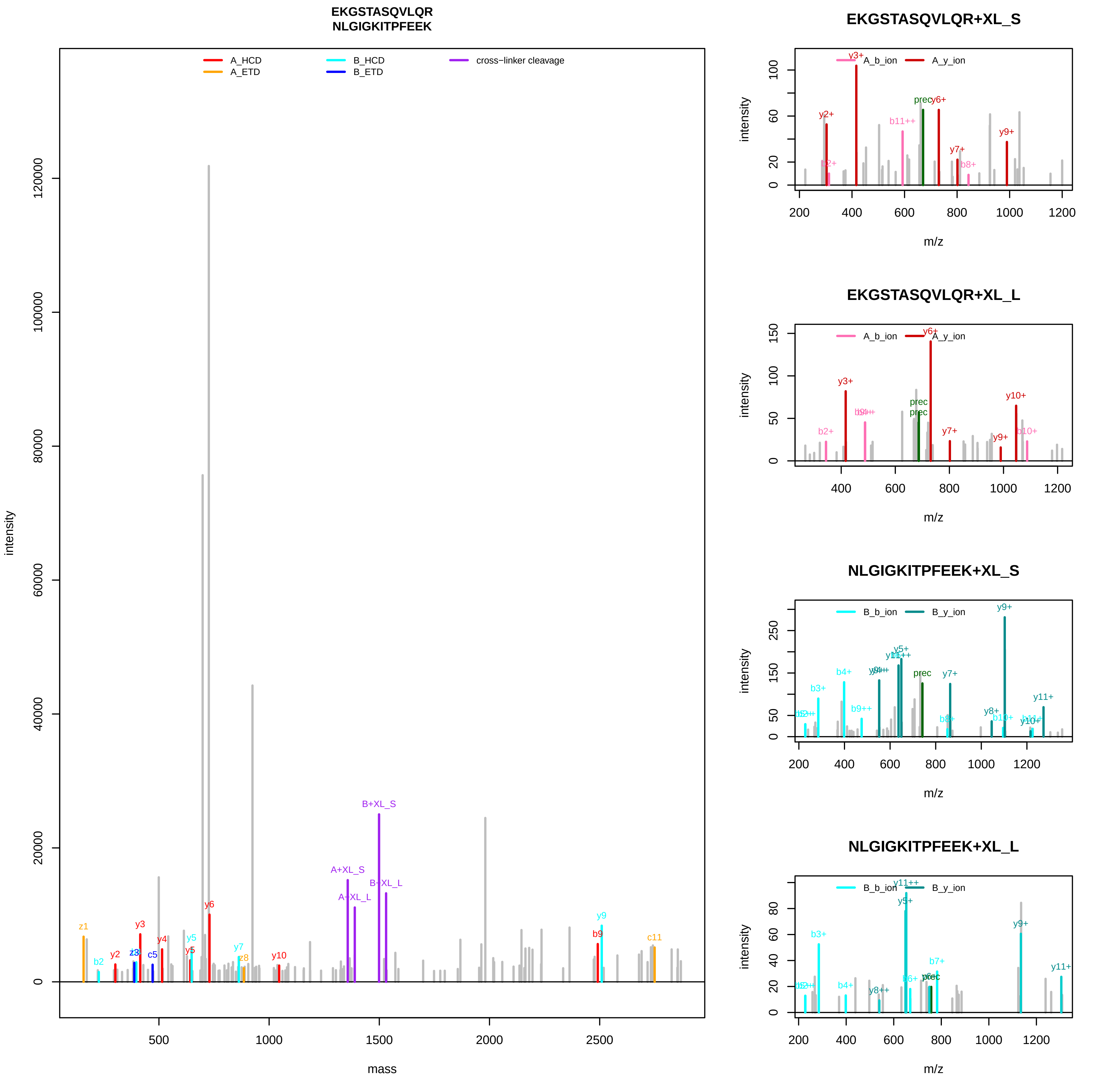

EKGSTASQVLQR
NLGIGKITPFEEK
A_HCD
A_ETD
B_HCD
B_ETD
cross−linker cleavage
intensity
mass
0
20000
40000
60000
80000
100000
120000
500
1000
1500
2000
2500
z1
b2
y2
y3
z3
c5
y4
y5
y6
y7
z8
y10
A+XL_S
A+XL_L
B+XL_S
B+XL_L
b9
y9
c11
EKGSTASQVLQR+XL_S
EKGSTASQVLQR+XL_L
NLGIGKITPFEEK+XL_S
NLGIGKITPFEEK+XL_L
A_b_ion
A_y_ion
B_b_ion
B_y_ion
m/z
intensity
prec
y2+
y3+
y6+
y7+
y9+
y10+
b2+
b8+
b10+
b11++
b3+
b4+
b7+
b8+
b9++
y5+
y7+
y8+
y8++
y9+
y11+
y11++
